# Population Structure of *Morpho helenor peleides* (Kollar, 1850) (Lepidoptera: Nymphalidae) Under Different Land Uses in the Caribbean Region of Colombia

**DOI:** 10.3390/insects16121243

**Published:** 2025-12-09

**Authors:** Carlos Elías Altamar-Bolívar, Juan David González-Trujillo, Luis G. Quijano-Cuervo, María Inés Moreno-Pallares, Neis José Martínez-Hernández

**Affiliations:** 1 Semillero de Investigación NEOPTERA, Grupo de Investigación Biodiversidad del Caribe Colombiano, Facultad de Ciencias Básicas, Universidad del Atlántico, Puerto Colombia 081007, Colombia; mariainesmoreno@mail.uniatlantico.edu.co; 2 Departamento de Biología, Facultad de Ciencias Exactas, Universidad Nacional de Colombia, Bogotá 110111, Colombia; jdgonzalezt@unal.edu.co; 3 Instituto de Investigaciones en Ecosistemas y Sustentabilidad, Universidad Nacional Autónoma de México (UNAM), Morelia 58000, Mexico; luisquijanocuervo@gmail.com

**Keywords:** population ecology, Lepidoptera, land use, population structure, POPAN

## Abstract

*Morpho* butterflies have the potential to act as a bioindicator species, since they are sensitive to changes in land uses and forest degradation; there have been few population studies of butterflies in the Caribbean region of Colombia. In this study, we aimed to analyze and characterize the population structure of *M. helenor peleides* and evaluate its variation across different land uses. We used the capture-mark and release technique, with 50 VanSomeren–Rydon type traps distributed in five sampling units with different land uses (forest, restoration areas, and pasture areas); temperature and luminosity were also measured. Butterflies were wing-marked and subsequently released. Sampling was carried out between June and September 2023. Jolly–Seber population models were constructed using the R code to obtain population size (*N*_i_), survival rate, and recruitment; also, other parameters were analyzed (abundance of the imagines, average sex ratio-ASR, displacement, permanence, and age). A total of 876 butterflies were tagged and released, and 33.7% were recaptured. Butterfly abundance was concentrated in conserved sampling units in the forest. The *N*_i_ ranged from 25 to 845 individuals within the population, and individual displacement and permanence were restricted in the forest, ASR was significantly male-skewed. The land use directly influenced the population structure of *M. helenor peleides*, suggesting that conserved areas are key to population persistence.

## Simple Summary

*Morpho* butterflies are known to be a charismatic species with large size and an iridescent blue colouring of the wings attracting collectors from all over the world. In this study, we aimed to analyze the population of *M. helenor peleides* and evaluate its variation across different areas. We used the capture-mark and release technique, with 50 traps distributed in different sites of a protected area in the Caribbean region of Colombia; temperature and luminosity were also measured. Butterflies were wing-marked and subsequently released. Sampling was carried out between June and September 2023. Demographic parameters like sex, age, abundance, displacement, and permanence time were analyzed. A total of 876 butterflies were tagged and released, and 33.7% were recaptured. Butterfly abundance was concentrated in the conserved site of the protected area, specifically in the forest interior. The population size ranged from 8 to 650 individuals within the population, and individual displacement was restricted between the forestry areas. The population was significantly male-skewed. The land use directly influenced the population structure of *M. helenor peleides*, suggesting that most conserved areas are key to population persistence and that behaviour and life history play an important role in the results observed.

## 1. Introduction

A population is a group of individuals of the same species interacting within the same space. The survival of a population is determined by the way those individuals interact with each other and with their surrounding environment [1]. Therefore, in order to ensure the permanence of animal populations of interest, it is necessary to know their status. As a tool for objectively studying populations, population ecologists rely on a series of statistical measures, known as demographic parameters, to describe that population [2,3].

This approach analyses adult demography within a population and examines how these parameters respond to the biotic and abiotic characteristics of the habitat [4,5,6]. Habitat alteration, such as fragmentation and land use changes, modify microclimatic conditions and the availability of nutritional and spatial resources for individuals [7,8,9,10]. These alterations can affect reproductive events and prevent species from reaching their optimal population size (population structure), thereby limiting the future resilience of populations [11,12].

Land use refers to the sum of human activities and arrangements that aim to utilize ecosystem services on the terrestrial surface. It is a significant factor in the interactions between society and nature and a major driver of global environmental change [12]. Land use changes occur constantly and at many scales and can have specific and cumulative effects on wildlife habitats [13]. Disturbances, including land use change, modify vegetation structure and can affect the population abundance and distribution of sensitive species, which therefore have different population dynamics under different land uses, including semi-natural areas and forest edges [14,15]. For example, frugivorous butterflies which depend on plants for nutritional resources in both their larval and adult stages, are especially reliant on the availability of suitable habitat to complete their life cycles and persist over time [10,16]. Habitat alterations driven by land use highlight the importance of assessing the responses of different taxa to changes in landscape structure, especially those with complex life cycles and specialized ecological requirements, such as butterflies.

Butterflies are highly sensitive to spatial and temporal habitat and microclimatic changes [8]. This, combined with their wide dispersal capacity, makes them an interesting model for population studies, as individual movements within an ecosystem have a significant impact on the observed population structure [16]. One of the most common butterfly groups in tropical forests are fruit-feeding butterflies, which are specialists feeding on decomposing fruits found on the forest floor [17]. Fruit-feeding butterflies have been used as bioindicators of ecosystem health and progress in ecological restoration projects in various ecosystems worldwide due to their sensitivity to habitat alterations, seasonal climatic changes, and land use changes [18,19,20]. *Morpho* (Fabricius, 1807) is a genus of fruit-feeding butterflies belonging to the family Nymphalidae, comprising more than 36 species in the Neotropical region [20]. In Colombia, the genus is represented by 14 species and 25 subspecies [21,22], most of which inhabit terrestrial ecosystems from sea level up to 2800 mamsl [23,24]. The subspecies *Morpho helenor peleides* (Kollar, 1850) is distributed across Northern Colombia, including the Bajo Cauca, Upper and Middle Magdalena, the Sierra Nevada de Santa Marta, and the Serranía de Perijá [24,25]. It is frequently found in fragments of Tropical Dry Forest (TDF), where it is considered a bioindicator of good forest conservation status [26,27,28]. However, given the current state of fragmentation of the TDF in Colombia—where less than 8% of the original forest cover remains—and the expansion of agricultural and livestock frontiers in the Caribbean lowlands [29,30,31], population studies are necessary to understand how these butterfly populations respond to land use changes (abundance, ASR, age, and permanence time) and how individuals move across different areas of forest fragments.

This study aimed to analyze the population structure of *M. h. peleides* and evaluate its variation across different land uses. By examining key demographic parameters and displacement patterns in response to habitat characteristics, we sought to improve our understanding of how land use changes affect this species, providing insights that will be relevant to biodiversity conservation and landscape management in the region.

## 2. Materials and Methods

### 2.1. Study Area

**Figure 1:**
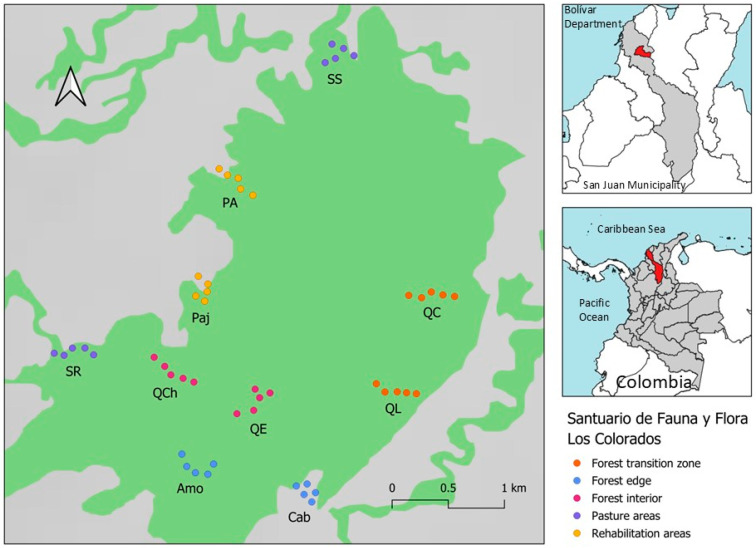
Location of sampling stations for CMR of *Morpho helenor peleides* in ‘Santuario de Flora y Fauna Los Colorados’, San Juan Nepomuceno municipality, Bolívar department, Colombia. *SS = San Salvador station, PAR = Puerto Arturo station, Paj = El Pajonal station, SR = Santa Rosa station, QCh = El Salto del Chivo station, QE = Quebrada La Escondida station, Amo = Amortiguación station, Cab = Cabaña station, QL = Quebrada Limón station, QC = Quebrada La Chana station*.

El Santuario de Flora y Fauna “Los Colorados” (SFFC) is a national natural park of Colombia (PNNC) with an area of 1000 hectares, located on the “Los Colorados” hill (9°54′ N 75°7′ W). This National Park is in the Bolívar department, municipality San Juan Nepomuceno, and is one of the most important remnants of the TDF in the foothills of the San Jacinto Mountain range [31]. The TDF is one of the most threatened ecosystems on the planet, and in Colombia, it is one of the least fragmented and degraded by illegal practices such as low-scale mining and land use changes [31,32,33]. The annual average temperature and precipitation of the TDF are 27 °C and 1500 mm, respectively, with interannual variation in the rainfall regime [32,34] (Figure 1).

SFFC is in the D2 climatic subunit, which means that the predominant climate is semi-humid with a bimodal rainfall regime, consisting of a very intense dry period between December and April and a less dry period between July and August; maximum rainfall occurs between October and November. During the three or four dry months, there is a deficit of available water (December to March). The vegetation in the SFFC is divided into three basic types: deciduous (loses leaves in the dry season), sub-hygrophilous (tolerates moisture changes without losing leaves), and hygrophilous (has sufficient moisture throughout the year) [34].

### 2.2. Field Work

#### 2.2.1. Capture-Mark and Release (CMR)

Ten sampling stations were established within the SFFC to characterize the population structure of *M. h. peleides* and their movement patterns. Each sampling station consisted of five Van Someren–Rydon (VSR) type traps hung 1.0 to 1.5 m above the ground, maintaining an average distance of 98 m (±25 m) between traps. The attractant consisted of a mixture of fermented fruits, panela (brown cane sugar), and vanilla essence because the adults usually feed on moist soil, animal droppings, and especially decomposed fruit [35]. Male butterflies of *M. helenor peleides* are very territorial and patrol along rivers and streams, while females are more elusive and fly in the canopy and undergrowth in search of egg-laying sites [36]. Host plants include several families such as Arecaceae, Bignoniaceae, Fabaceae, Mimosaceae, Ochnaceae, Poaceae, and Sapindaceae [37]. Vouchers of this subspecies are deposited at the insect collection of the Ecology and Entomology Lab of Universidad del Atlántico (female, UA: UARC: LEP00412 and male, UA: UARC: LEP00437).

The network of stations covered three different land uses: forest (F), restoration areas (RA), and pasture areas (PZ). The forest land use was divided into three zones, forest interior (FI), forest transition zone (TZ), and forest edge (FE). In total, five different sampling units were selected for analyses (Table A1). To characterize the probability of occurrence and movements within the fragment, the capture-mark-recapture technique was used. This involved marking captured individuals with a micro-point marker (0.5) on the ventral side of the forewings with a unique alphanumeric code (ID) during each sampling, then releasing them. Five (5) capture samplings were conducted, with an average frequency of 16 days, between June and September 2023, for a total of 15 CMR events (Table A2). Each sampling lasted seven days, of which three were for the CMR of butterflies; each sample had three CMR events: at 24, 72, and 120 h. CMR sampling took place between 7 and 14 h, requiring two teams of four people to complete the fieldwork (effort of 162 man/h). One of the groups dealt with the points concerning forest land use, while the second group dealt with the stations in pasture and restoration areas.

In each CMR sampling, the following were recorded in situ: sampling number, date, ID, sex, and age category; young (“A”: butterflies in an evident state of recent emergence without any wing damage), intermediate (“B”: butterflies with wings in good condition with descaled regions), and old (“C”: butterflies with descaled and/or fragmented wings) [35,36].

#### 2.2.2. Environmental Variables

Temperature and humidity were measured in the study area with nine data loggers (Extech©, RHT50 datalogger, Extech Instruments, Nashua, NH, USA), installed in all stations, covering every land use category in the landscape. The data loggers were programmed to record the variables every 30 min and remained in the field throughout the whole sampling period. Luminosity (Extech©, LT300 Lux Meter Extech Instruments, Nashua, NH, USA) was measured during the CMR events for all stations.

### 2.3. Data Analysis

#### 2.3.1. Population Structure

Population sizes were estimated in the framework of constrained linear models (CLM), which apply the methodology of generalized linear models to mark-recapture data [2]. The POPAN parameterization of the Jolly–Seber approach is suitable for open populations with time-varying deaths and recruitment. POPAN estimates three primary parameters: daily survival rates (*Φ*i; staying at a site, combines mortality and emigration), daily catchability, *p*_i_, and daily recruitment, calculated as the percentage entering the population, *p_ent_*_i_. These are then used to derive the parameters of total daily recruitment, *β*_i_, daily population size, *N*_i_, and total population *N*. We modelled the phi and *p_ent_* parameter as constant in time and sex (.), sex-dependent (g), time-dependent in a factorial (t), linear (T), quadratic (T2) or cubic (T3) manners, and with additive (g + t, g + T, g + T2…) or interactive (g·t,g·T,g·T2…) response to time and sex. The parameter *p* was kept as a constant to meet the Jolly–Seber assumption that all individuals have the same probability of being captured and *N_sup_* was kept as a constant to avoid illogical estimates; package RMark v 3.0.0. was used for the construction of models. To select the best model, were used the Akaike information criterion (AIC) and the degree of fit (w) [37,38,39,40,41,42]. The results and discussion are focused on the maximum and minimum values of the estimates, highlighting the possible causes of variation in these values.

The adult sex ratio (ASR) was calculated considering only adults [43]:

(1) male/male+female

where the resulting value of the ASR is 0.5 if the number of males and females is equal. This method is the most recommended for estimating sex ratios in nature, given that it considers individuals as discrete units and thereby reflects the relative abundance of each sex in a population. We used chi-square (X2) tests to determine if the observed ASR statistically differed [44]. To analyze the fluctuation of age structure across samples, 100% shadow plots were constructed in R, and we used recapture records to estimate the persistence time (days) in the sampling units. The displacement analysis was obtained from the recapture record, and the shortest linear trajectories between each trap-core were used to obtain the distance travelled (in metres) between capture and recapture. Recaptures in the same trap were considered as a minimum effective displacement of 10 m. Analyses were performed using QGIS 3.3v software [45]. A regression analysis was carried out to analyze whether or not there are significant differences in the distances travelled between males and females in R code.

#### 2.3.2. Abundance and Environmental Variables

Generalized linear models (GLM) were constructed to determine the effect of the two explanatory variables (temperature and luminosity) and the sampling units (samU) on the total abundance of *M. h. peleides* (response variable). For this purpose, a model with Poisson error distribution (‘log’ link function) was established for abundance, considering its nature as a discrete count variable. To select the minimum appropriate model, we compared all different combinations of variables and their interactions with a null model. These models were built using the packages lme4 [46] and MuMin [47] in R. From the models built, we selected the model with the lowest Akaike criterion value (AIC), including the null model [41,42,43,44]. Finally, ANOVA were constructed to observe the significance of the combinations of factors on the response variable [41].

## 3. Results

### 3.1. Population Structure

#### 3.1.1. Abundance and Population Size

A total of 876 butterflies (net abundance) were captured and released (♂: 563; ♀: 313), 296 of which (33.78%) were recaptured (including recaptures during the same CMR event and between different CMR events). The CMR events with the highest number of captures occurred at the end of July (21–23 July). Abundance tended to increase towards the end of this month, followed by a decline until August and early September (Table 1).

The population model that best explained the distribution of the data was one in which Ph i (t) varies over time (t) and *p_ent_* (g × t) varies with sex (g) and time (t), and includes their interaction (Table 2).

**Figure 2:**
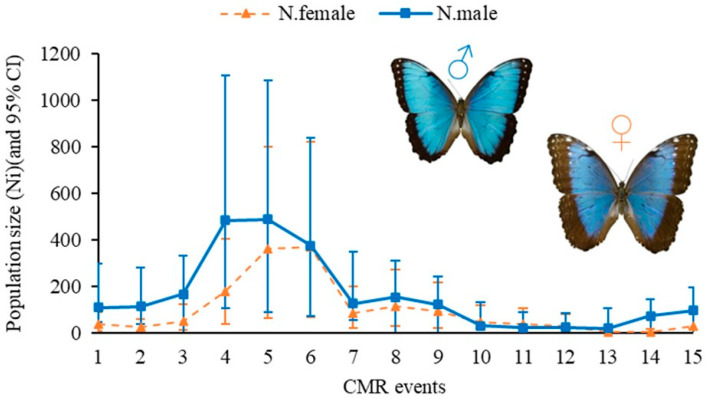
Estimated population size (*N*_i_) using Jolly–Seber (POPAN) for the *Morpho helenor peleides* butterfly in Santuario de Flora y Fauna Los Colorados, San Juan Nepomuceno municipality, Bolivar Department, Colombia.

The estimated *N_gross_* according to this JS-POPAN model was 1015 females and 1577 males in the study area, resulting in a density of 2.59 adults per hectare. The selected model forecasted a range of 25–848 individuals in the population (*N*_i_), with the largest projected population size in July and the smallest in September. The largest population increase occurred at the end of July 2023, followed by a second peak in early August (Figure 2).

**Figure 3:**
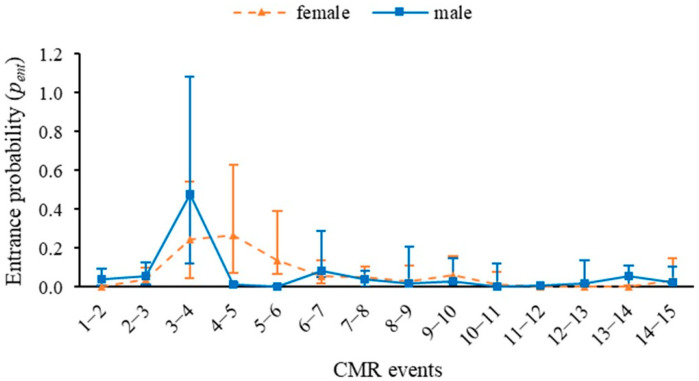
Entrance probability of JS-POPAN for *Morpho helenor peleides* population based on 15 mark-recapture events over four months (June–September 2023) in Santuario de Flora y Fauna Los Colorados, San Juan Nepomuceno municipality, Bolívar Department, Colombia.

The probability of entry of new individuals (*p_ent_*) for females was the highest probability of entry, 27%, while for males it reached 47% for the same date, at the beginning of July 2023 (Figure 3). The overall probability of capture was 0.3 ± 0.008 (0.283; 0.318).

**Figure 4:**
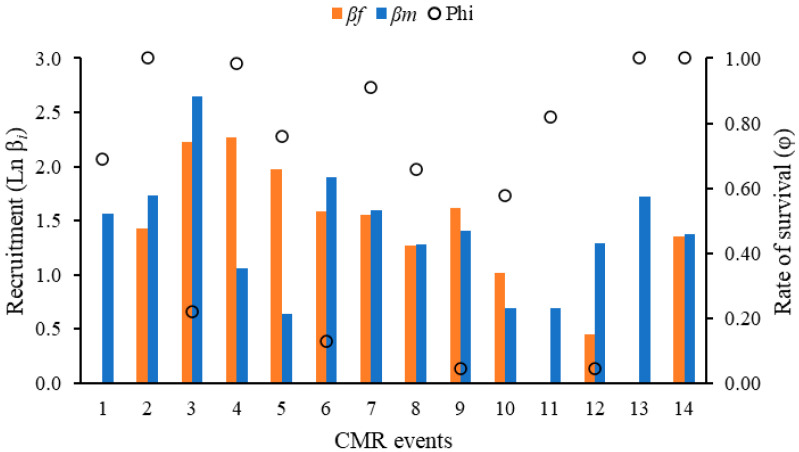
Recruitment for females (*β*_f_) and males (*β*_m_), and survival rate (*Φ*_i_) estimated for the *Morpho helenor peleides* population based on 15 mark-recapture events over four months (June–September 2023) in Santuario de Flora y Fauna Los Colorados, San Juan Nepomuceno municipality, Bolívar Department, Colombia. Recruitment (*β*_i_) was transformed by the natural logarithm (Ln *β*_i_) to facilitate comparison.

The *β*_i_ and *Φ*_i_ showed a similar pattern of increase and decrease to the *N*_i_; recruitment was highest at the beginning of July and decreased towards August and September, when male values exceeded female values (Figure 4).

#### 3.1.2. Average Sex Ratio (ASR)

**Figure 5:**
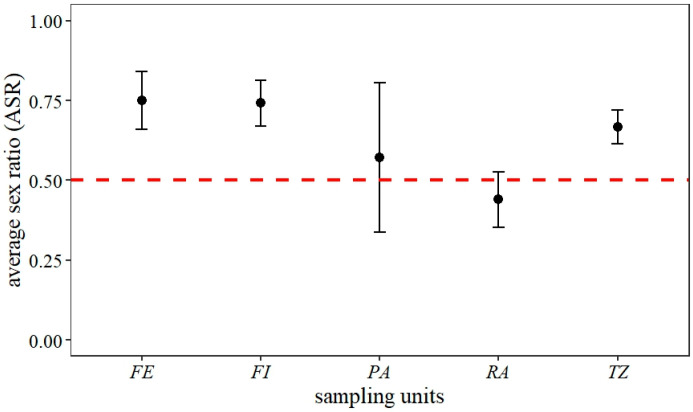
Average sex ratio (ASR) of *Morpho helenor peleides* population based on 15 mark-recapture events over four months (June–September 2023) in Santuario de Flora y Fauna Los Colorados, San Juan Nepomuceno municipality, Bolívar Department, Colombia. *FE = forest edge, FI = forest interior, PA = pasture areas, RA = restoration areas, TZ = forest transition zone*.

The sex ratio fluctuated between 0.4 and 1.0 across each capture event. The overall results showed a significantly male-skewed sex ratio (*p* < 0.05, df = 14, X^2^ = 63.44). Separating the sex ratio by land use, in the three forest zone categories, the sex ratio was skewed towards males, while in less conserved land use areas (restoration areas and pasture areas), the observed sex ratio was close to 0.5 (Figure 5).

#### 3.1.3. Age Structure

**Figure 6:**
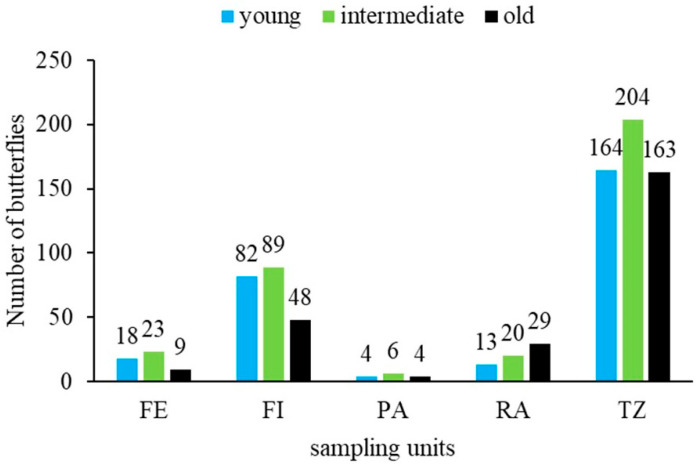
Age structure of the *Morpho helenor peleides* population based on 15 mark-recapture events over four months (June–September 2023) in Santuario de Flora y Fauna Los Colorados, San Juan Nepomuceno municipality, Bolívar Department, Colombia. *FE = forest edge, FI = forest interior, PA = pasture areas, RA = restoration areas, TZ = forest transition zone*.

The behaviour of the recorded age stages or phases of the captured butterflies showed two population emergence peaks of young butterflies at the beginning of July and in September, with a higher presence of intermediate and old butterflies at the end of July and August. In terms of sampling units, young and intermediate butterflies were more abundant in the forest interior, forest transition zones, and forest edge, while older butterflies were most abundant in restoration areas (Figure 6).

#### 3.1.4. Permanence Time

Based on the recapture analysis, there were 296 recaptures (♂, 199; ♀, 97), with a minimum residence time of two days for both females and males. Regarding the maximum residence time, males reached just over a month (34 days), while females’ maximum residence time was 23 days. The most frequent residence time for both sexes was between 2 and 5.5 days. When dividing by sampling units, the average permanence times of *M. h. peleides* were highest in forest interior zones (11.5 days) and lowest in forest edge areas (2 days) (Table 3).

#### 3.1.5. Displacement

The displacement of butterflies was restricted to the forest interior and transition zones. Movement between these zones was much more frequent than between other natural park areas, so forest was the land use with most recapture records. The least frequent movements occurred between forest interior and pasture areas, forest interior and restoration areas, forest edge and transition zones, and transition zones and pasture areas (Table 4). Regarding sex and distance travelled (m), significant differences were found between males and females (*p* = 0.0239, R^2^ = 0.017, F-statistic = 5.156). However, the maximum distance travelled for males was 1.9 Km and females was 2.9 Km.

### 3.2. Abundance and Environmental Variables

#### 3.2.1. Temperature, Humidity, and Light Intensity

Regarding the behaviour of temperature and relative humidity, the highest average values measured during the entire study period were reached in mid-July and September, while humidity tended to increase at the beginning of the sampling period in June and in August, coinciding with the arrival of rains in the study area and the warmer period known as “Veranillo de San Juan” in July (Table A3).

#### 3.2.2. Influence of Environmental Variables

The generalized linear model that provided the best explanation had an AIC value of 1291.762, while the null model yielded a value of 1784.053 (Table 5).

The variation in abundance was affected by the sampling unit (samU) of the areas as well as changes in ambient temperature (AT) and light intensity (LUX) (Table 6).

## 4. Discussion

The main findings of this research show that population parameters such as abundance, sex ratio, displacement trajectories, and age ratio in *M. h. peleides* respond not only to different land uses but also to different areas within the forest, which highlights the bioindicator role of the species. The behaviour of population size, probability of survival, and *p*_ent_ highlight the role of SFFC as a key protected environment in the refuge of *M. h. peleides*, so the determination that interior forest areas are more beneficial for the populations of this species is valuable information for the development of conservation plans in the TDF, and reaffirms the necessity to declare more natural protected areas around the world, because although in intermediate vegetation cover or secondary forests the diversity of fruit-feeding butterflies is high, some species of fruit-feeding butterflies exhibit specialist habitat requirements [48,49]. Therefore, changes in land use and fragmentation of habitats are a primary driver of biodiversity loss [50], particularly in tropical regions, where deforestation and land use change continue to accelerate [51,52].

The abundance of *M. h. peleides* recorded in this study was higher than those described by other population studies of species within this genus in Colombia. For example, the studies of Marquez-P and Martinez-H [27] with the same subspecies, in a nearby locality, with the same CMR technique and with a greater number of samplings, recorded 287 adults, while Ochoa et al. [53] who studied the species *Morpho rhodopteron* (Godman and Salvin, 1880) in the Sierra Nevada de Santa Marta using insect nets, recorded 115 adults and Prieto et al. [54] who studied *Morpho sulkowskyi* (Kollar, 1850) in the Andean forests of Cauca also using an insect net, recorded a total of 152 adults. In the aforementioned works, recapture percentages were around 20%, so it is inferred that in this work the sampling effort was optimal for the population studied, and we consider that the results of the research reflect the reality of the population size of *M. h. peleides* in “Los Colorados” (25–848 adults). These results also show that SFFC’s TDF areas contain one of the most numerous populations of *M. h. peleides* in the Colombian Caribbean, so the fact that it is a protected area contributes to the persistence of the population.

The fluctuation of population size (*N*_i_) throughout CMR events is probably related to the biology of butterflies and their heliothermal condition; higher butterfly activity translates into greater net population size during events with especially high temperature and luminosity [9,10,48,49]. This particularity of butterflies’ biology supports MGL’s significant results. This same pattern of behaviour is applied to the *Φ*_i_ and *β*_i_ parameters, showing that climatic events can modulate the population parameters evaluated. However, these longer periods of *Morpho helenor peleides* activity are likely also influenced by the emergence of adults at these times of the year, a first event in July and another in August, as suggested by fluctuations in population size.

The mean *p_ent_* parameter was low for both sexes (♀6.7% and ♂6.2%), which is directly related to the trajectories of *M. h. peleides* analyses. This may indicate that the individuals of the species stay mainly in the internal areas of the fragment (Forest land use) and migrate low to the external and degraded areas of the park, which are also under constant human influence through the livestock practices of these areas and the processes of plant degradation that are observed in the restoration areas, due to *M. h. peleides* preferring forest zones and secondary forest [27]. The results suggest that females are more likely to be the ones entering or leaving the population.

In this study, males were more abundant than females; this has also been reported in other population studies of *Morpho* in Colombia [28,46,47]. The male-skewed ASR of *M. h. peleides* in this study is related to behavioural differences between the sexes. Male *Morpho* exhibit territorial behaviour and continuously patrol rivers and streams [28,50,51]. Meanwhile, females remain hidden within the foliage (even during feeding hours) except when they are flying over the understory in search of potential host plants for their eggs [27,52,53]. Female *M. h. peleides* lay only one egg per plant, which favours larval survival by avoiding competition and could also explain the shorter residence times and more distance travelled for females than for males, suggesting that in this type of butterfly, there is strong genetic programming that regulates their behaviour and shapes the functioning of populations, and which is observed in several fruit butterfly communities in South America, Asia, and Africa [48,49,53,54,55,56,57,58,59,60].

Regarding the distribution of *M. h. peleides* age categories, the young and intermediate categories were more abundant in areas with foliage that are likely to provide the butterflies with safer sites, where young butterflies take refuge from the attack of possible predators; this could also be because these are the sites where larvae and pupae are concentrated because the environmental conditions are optimal [9,54,55,56]. These results, together with the observed permanence times, suggest that butterflies are not only more abundant within more conserved areas of the forest, but also stay longer in them and move more frequently between forested areas, confirming that the biology of butterfly species is also a strong regulator of observed population structure along with dispersal ability that shapes several of their observed demographic parameters [57,58,59,60,61].

## 5. Conclusions

In conclusion, population parameters like abundance, population size, ASR, displacement patterns, and age structure in the *M. h. peleides* population of the SFFC differ among land uses and among different categories of forest cover, likely due to a combination of biological and ecological factors. This highlights the bioindicator role of the species and their preference for forest interior areas and forest transition zones, a characteristic of the species of the genus that has been mentioned by other authors but that had not been analyzed in depth with a quantitative approach including the variables analyzed in this work.

## Figures and Tables

**Table 1:** Abundance of *Morpho helenor peleides* population based on 15 mark-recapture events over five CMR events (June–September 2023) in Santuario de Flora y Fauna Los Colorados, San Juan Nepomuceno municipality, Bolívar Department, Colombia. SU = sampling units, *FE = forest edge, FI = forest interior, PA = pasture areas, RA = restoration areas, TZ = forest transition zone*.

SU	1	2	3	4	5	Total
FE	4 (4♂:0♀)	20 (14♂:6♀)	4 (3♂:1♀)	3 (2♂:1♀)	6 (4♂:2♀)	37 (27♂:10♀)
	0	9 (3♂:6♀)	2 (2♂:0♀)	1 (0♂:1♀)	1 (1♂:0♀)	13 (6♂:7♀)
FI	11 (9♂:2♀)	22 (17♂:15♀)	7 (4♂:3♀)	1 (0♂:1♀)	11 (10♂:1♀)	62 (40♂:22♀)
	2 (2♂:0♀)	103 (65♂:38♀)	31 (23♂:8♀)	4 (3♂:1♀)	17 (16♂:1♀)	157 (109♂:55♀)
PA	1 (1♂:0♀)	0	0	0	0	1 (1♂:0♀)
	0	12 (6♂:6♀)	0	0	1 (1♂:0♀)	13 (7♂:6♀)
RA	1 (0♂:1♀)	21 (9♂:12♀)	10 (7♂:3♀)	1 (1♂:0♀)	5 (4♂:1♀)	38 (21♂:17♀)
	1 (0♂:1♀)	11 (3♂:8♀)	3 (2♂:1♀)	2 (0♂:2♀)	7 (5♂:2♀)	24 (10♂:14♀)
TZ	51 (38♂:13♀)	146 (100♂:46♀)	40 (20♂:20♀)	17 (6♂:11♀)	4 (4♂:0♀)	258 (168♂:90♀)
	57 (48♂:9♀)	156 (90♂:66♀)	39 (23♂:16♀)	14 (7♂:7♀)	7 (6♂:1♀)	273 (174♂:99♀)
total	128 (102♂:26♀)	510 (307♂:203♀)	136 (84♂:52♀)	43 (19♂:23♀)	59 (51♂:8♀)	876 (563♂:313♀)

**Table 2:** The best performing JS-POPAN models for the *Morpho helenor peleides* population based on 15 mark-recapture events over four months (June–September 2023) in *Santuario de Flora y Fauna Los Colorados*, San Juan Nepomuceno municipality, Bolívar Department, Colombia. *K = number of parameters of model, AICc = Akaike information criterion, ΔAICc = Akaike information criterion corrected, w = model weight*.

No.	Model	npar	AICc	ΔAICc	w	Deviance
59	φ(t)*p*(.)*p*_ent_(g × t)*N*_sup_(.)	44	1810.42	0	1.00	−3546.783
63	φ(t)*p*(.)*p*_ent_(g + t)*N*_sup_(.)	31	1852.35	41.92748	0.00	−3477.07
70	φ(t)*p*(.)*p*_ent_(t)*N*_sup_(.)	30	1864.99	54.56469	0.00	−3462.321
17	φ(T)*p*(.)*p*_ent_(g × t)*N*_sup_(.)	32	1956.32	145.8997	0.00	−3375.212
31	φ(T^2^)*p*(.)*p*_ent_(g × t)*N*_sup_(.)	32	1956.32	145.8997	0.00	−3375.212
45	φ(T^3^)*p*(.)*p*_ent_(g × t)*N*_sup_(.)	32	1956.32	145.8997	0.00	−3375.212
3	φ(.)*p*(.)*p*_ent_(g × t)*N*_sup_(.)	31	1974.72	164.30008	0.00	−3354.697
21	φ(T)*p*(.)*p*_ent_(g + t)*N*_sup_(.)	19	1986.39	175.96456	0.00	−3317.944
1	φ(.)*p*(.)*p*_ent_(.)*N*_sup_(.)	4	2718.48	908.05814	0.00	−2555.221

**Table 3:** Permanence time of *Morpho helenor peleides* population based on 15 mark-recapture events over four months (June–September 2023) in Santuario de Flora y Fauna Los Colorados, San Juan Nepomuceno municipality, Bolívar Department, Colombia. *FE = forest edge, FI = forest interior, PA = pasture areas, RA = restoration areas, TZ = forest transition zone, QCh = quebrada La Chana, QLE = quebrada La Escondida, QC = quebrada La Chana, QL = quebrada El Limón, Am = Amortiguación, Paj = El Pajonal, PAR = Puerto Arturo, SR = Santa Rosa*.

Sampling Units	Station	Permanence (days)
FE	Am	2♀
		2♂
FI	QCh	3♀
		11.5♂
	QLE	2.57♀
		4.46♂
PA	SR	7.67♀
		4♂
RA	Paj	3♀
		2.67♂
	PAR	2♀
		2.5♂
TZ	QC	5.39♀
		5.63♂
	QL	4.54♀
		7.38♂

**Table 4:** Displacement trajectories recorded within and across sampling units based on the 296 recaptures records of *Morpho helenor peleides* population during 15 mark-recapture events over four months (June–September 2023) in Santuario de Flora y Fauna Los Colorados, San Juan Nepomuceno municipality, Bolívar Department, Colombia. *FI = forest interior, TZ = forest transition zone, FE = edge, RA = restoration areas, PA = pasture areas*.

Trajectories	Female	Male	Mean	SD	Sum
limited FI	10	43	26.5	23.33	53
limited FE	1	2	1.5	0.71	3
limited TZ	69	135	102	46.67	204
limited PA	3	1	2	1.41	4
limited RA	4	7	5.5	2.12	11
across FI-FE	0	3	1.5	2.12	3
across FI-TZ	7	5	6	1.41	12
across FI-PA	0	1	0.5	0.71	1
across FI-RA	0	1	0.5	0.71	1
across FE-TZ	0	1	0.5	0.71	1
across FE-PA	0	0	0	0.00	0
across FE-RA	0	0	0	0.00	0
across TZ-PA	1	0	0.5	0.71	1
across TZ-RA	2	0	1	1.41	2
across RZ-PA	0	0	0	0.00	0

**Table 5:** Best generalized linear models explaining the abundance of *Morpho helenor peleides* population as a function of sampling location and environmental variables during 15 mark-recapture events over four months (June–September 2023) in Santuario de Flora y Fauna Los Colorados, San Juan Nepomuceno municipality, Bolívar Department, Colombia. *K = numbers of parameters, logLik = logarithm of plausibility, AIC = Akaike information criterion, ΔAIC = Akaike delta information criterion, w = model weight, samU = sampling units, LUX = luminosity, T = temperature*.

No.	Models	K	logLik	AIC	ΔAIC	w
128	Mhp~ samU + LUX + T + samU × LUX + samU × T + LUX × T + samU × LUX × T	8	−637.881	1291.762	0.000	9.70 × 10^−1^
16	Mhp~ samU + LUX + T + samU × LUX	5	−645.242	1300.483	8.721	1.24 × 10^−2^
32	Mhp~ samU + LUX + T + samU × LUX + samU × T	6	−644.822	1301.643	9.881	6.94 × 10^−3^
48	Mhp~ samU + LUX + T + samU × LUX + LUX × T	6	−645.121	1302.243	10.481	5.14 × 10^−3^
64	Mhp~ samU + LUX + T + samU × LUX + samU × TA + LUX × T	7	−644.350	1302.701	10.939	4.09 × 10^−3^
1	Mhp~ null	1	−891.026	1784.053	492.290	1.22 × 10^−17^

**Table 6:** Type 2 test regression table (ANOVA) of the generalized linear model that best explained the variability in *Morpho helenor peleides* abundance (see Table 5) based on 15 mark-recapture events over four months (June–September 2023) in *Santuario de Flora y Fauna Los Colorados*, San Juan Nepomuceno municipality, Bolívar Department, Colombia. *SU = sampling units*, *LUX = luminosity, T = temperature, lrX2 = likelihood ratio, df = degrees of freedom, Pr = significance level*.

Response Variable	Explanatory Variable	lr X2	df	Pr
*Butterfly abundance*	samU	132.478	1	**<2.2 × 10^−16^**
	LUX	52.275	1	**4.83 × 10^−13^**
	T	78.468	1	**<2.2 × 10^−16^**
	samU:LUX	7.492	1	**6.20 × 10^−3^**
	samU:T	1.542	1	0.214
	samU:T	0.942	1	0.3316571
	samU:LUX:T	12.939	1	**0.0003219**

bold indicates significance of Pr value.

## Data Availability

The data of this research is available for the community in Zenodo at https://doi.org/10.5281/zenodo.15636480.

## Abbreviations

The following abbreviations are used in this manuscript:

TDF	Tropical dry forest
F	Forest
FI	Forest interior
FZ	Forest transition zone
FB	Forest border
RA	Restoration areas
PA	Pasture areas

## Appendix A

### Appendix A.1

**Table A1:** Description of units and sampling stations selected for monitoring of the *Morpho helenor peleides* population based on 15 mark-recapture events over four months (June–September 2023) in *Santuario de Flora y Fauna Los Colorados*, San Juan Nepomuceno municipality, Bolívar Department, Colombia.

Sampling Units	Stations	Description
Forest interior (FI)	Quebradas (QCh) Chivos y La Escondida (QLE)	The forest interior zones of the fragment were characterized by having very little intervention from infrastructure and/or human activity, they are forested areas with trees over 20 m high and with little development of low vegetation strata. In these stations, the central trap location (number 3) is located more than 600 m from the edge forest line.
Transition forest zone (TZ)	Quebrada Chana (QC) y Limón (QL)	These zones correspond to areas where the traps are placed perpendicular to the edge forest line, approximately 700 m in length from the visible edge into the forest.
Forest edge (FE)	Caban (Ca) y Amortiguación (Am)	These zones correspond to places in the fragment where the continuity of the forest canopy cover and its vegetation development ends suddenly, cut with roads and human trails. Within this category are the hut and buffer stations bordering concrete roads and trails frequently used by vehicles and horse-drawn transport.
Restoration areas (RA)	Pajonal (Paj) y Puerto Arturo (PAR)	These areas correspond to places whose property title has recently been acquired by PNNC; therefore, they are places with a land use typical of intensive livestock activities and bordering the dry forest fragment; these places are in the process of restoration through the implementation of management plans that include the planting of native trees such as Carreto (*A. polyneurum*) and Guayacán (*B. arborea*).
Pasture areas (PA)	San Salvador (SS) y Rosa (SR)	These zones correspond to places where land conflict persists, i.e., areas adjacent to the forest fragment that belong to private properties that maintain land uses for livestock and grazing; these areas present permanent clearing activities.

**Table A2:** Date of sampling and events capture occasions of the *Morpho helenor peleides* population for 15 mark-recapture events over four months (June–September 2023) in Santuario de Flora y Fauna Los Colorados, San Juan Nepomuceno municipality, Bolívar Department, Colombia.

Date (2023)	Sampling	CMR Events
30 June	1	1
2 July		2
4 July		3
21 July	2	4
23 July		5
25 July		6
5 August	3	7
7 August		8
9 August		9
20 August	4	10
22 August		11
24 August		12
14 September	5	13
16 September		14
18 September		15

**Table A3:** Ambient temperature and humidity throughout the study of *Morpho helenor peleides* population based on 15 mark-recapture events over four months (June–September 2023) in *Santuario de Flora y Fauna Los Colorados*, San Juan Nepomuceno municipality, Bolívar Department, Colombia. Measurements were taken between June and September 2023. *Mean ± standard deviation (minimum; maximum)*.

Sample	Temperature (°C)	Humidity (%)	Luminosity (lux)
1	26.68 ± 1.96 (24.1; 31)	94.08 ± 4.13 (77.5; 98.1)	0.019 ± 0.009 (0.003; 0.034)
2	29.9 ± 3.17 (24.5; 40)	76.6 ± 12.22 (50.2; 93.4)	0.837 ± 0.514 (0.389; 1.797)
3	28.23 ± 2.32 (24.6; 32.5)	87.03 ± 7.72 (68.5; 96.9)	0.554 ± 0.476 (0.202; 1.542)
4	30.08 ± 2.04 (27.5; 34.6)	82.34 ± 6.29 (66.3; 93.1)	1.509 ± 1.317 (0.232; 4.364)
5	29.6 ± 0.098 (29.4; 29.7)	82.12 ± 0.61 (81.3; 83)	0.845 ± 0.718 (0.1; 2.287)
